# A NKG2D Thr72Ala polymorphism is not associated with HAM/TSP and proviral load values in Peruvian HTLV-1 infected patients with HAM/TSP and asymptomatic carriers

**DOI:** 10.1186/1742-4690-12-S1-O37

**Published:** 2015-08-28

**Authors:** Cynthia Bernia, Jason Rosado, Giovanni López, Eduardo Gotuzzo, Michael Talledo

**Affiliations:** 1 Inst de Med Trop Alexander von Humboldt, Univ. Peruana Cayetano Heredia, Lima, Peru

## Background

NKG2D is an activating receptor mainly expressed on Natural Killer (NK) cells, its ligands has an abnormal expression in virus infected cells and tumoral cells. The SNP rs2255336 G/A generates a substitution of alanine to threonine (NKG2D Thr72Ala) associated with a low and high cytotoxic activity of NK cells respectively. This study investigated the association between the NKG2D Thr72Ala polymorphism with HAM/TSP and PVL values in Peruvian HTLV-1 infected patients with HAM/TSP and asymptomatic carriers.

## Methods

215 Peruvian HTLV-1 infected patients (142 asymptomatic carriers (AC) and 73 HAM/TSP) were evaluated. NKG2D Thr72Ala polymorphism were genotyped by polymerase chain reaction using allele-specific primers, 37 ancestry informative markers (AIM) were genotyped to correct by population stratification using the first three principal components. Proviral load (PVL) was measured using the endogenous retrovirus 3 (ERV-3). Association of the NKG2D Thr72Ala polymorphism with HAM/TSP and PVL were performed by univariate analysis using Pearson's chi-square test and Mann-Whitney U-test and by multivariate analysis using logistic regression and linear regression analysis. STATA software was used for all analysis.

## Results

The NKG2D Thr72Ala polymorphism with G/G genotype showed a higher prevalence in asymptomatic carrier (64.13%) respect to HAM/TSP patients (34.87%), however these differences were not statistically significant (P>0.05). No association was found between the different genotypes of NKG2D Thr72Ala with PVL (P>0.05), or with the presence of HAM/TSP (P> 0.05). The assessment of the association between allele G (OR=2.3760; CI= 0.7744 to 7.2901) or the allele A (OR= 0.4209; CI=0.1372 to 1.2914) with the presence of HAM/TSP were not statistically significant (P>0.05).

## Conclusions

The results of this study indicate the need to evaluate the association of polymorphisms in NKG2D gene with HAM/TSP and PVL in other independent populations of HTLV-1 infected individuals in order to verify an association of disease state with host genetic.

